# Inferring microRNA-disease association by hybrid recommendation algorithm and unbalanced bi-random walk on heterogeneous network

**DOI:** 10.1038/s41598-019-39226-x

**Published:** 2019-02-21

**Authors:** Dong-Ling Yu, Yuan-Lin Ma, Zu-Guo Yu

**Affiliations:** 1 Key Laboratory of Intelligent Computing and Information Processing of Ministry of Education and Hunan Key Laboratory for Computation and Simulation in Science and Engineering, Xiangtan University, Xiangtan, Hunan 411105 P.R. China; 2 School of Electrical Engineering and Computer Science, Queensland University of Technology, Brisbane, Q4001 Australia

## Abstract

More and more research works have indicated that microRNAs (miRNAs) play indispensable roles in exploring the pathogenesis of diseases. Detecting miRNA-disease associations by experimental techniques in biology is expensive and time-consuming. Hence, it is important to propose reliable and accurate computational methods to exploring potential miRNAs related diseases. In our work, we develop a novel method (BRWHNHA) to uncover potential miRNAs associated with diseases based on hybrid recommendation algorithm and unbalanced bi-random walk. We first integrate the Gaussian interaction profile kernel similarity into the miRNA functional similarity network and the disease semantic similarity network. Then we calculate the transition probability matrix of bipartite network by using hybrid recommendation algorithm. Finally, we adopt unbalanced bi-random walk on the heterogeneous network to infer undiscovered miRNA-disease relationships. We tested BRWHNHA on 22 diseases based on five-fold cross-validation and achieves reliable performance with average AUC of 0.857, which an area under the ROC curve ranging from 0.807 to 0.924. As a result, BRWHNHA significantly improves the performance of inferring potential miRNA-disease association compared with previous methods. Moreover, the case studies on lung neoplasms and prostate neoplasms also illustrate that BRWHNHA is superior to previous prediction methods and is more advantageous in exploring potential miRNAs related diseases. All source codes can be downloaded from https://github.com/myl446/BRWHNHA.

## Introduction

MicroRNAs (miRNAs) are a class of short non–coding RNAs (21–25 nt)^1–3^. As an important transcriptional regulatory factor, miRNAs are widely involved in the biological procedures of disease-related gene regulation, which is closely related to human multi-gene diseases^4–7^. Increasing evidences have demonstrated that miRNAs play a critical role in the emergence and development of diseases^8,9^. Hence, revealing miRNAs associated diseases is an efficient way to accelerate the acquaintance about disease pathology at the molecular level^10–13^.

As detecting miRNA-disease associations by experimental techniques is expensive and time-consuming, many effective computational methods about the prediction of the relationship between miRNAs and diseases have been proposed. For example, Jiang *et al*.^14^ proposed a computational approach to infer potential miRNA-disease associations by hypergeometric distribution. For a given disease, they priorited the entire human miRNAs. In addition, Jiang *et al*.^15^ further improved the calculation of concordance score between a miRNA and a given disease. Chen *et al*.^16^ firstly presented a prediction computational method named RWRMDA based on global network similarity, to predict novel human miRNA-disease associations by adopting the method of random walk on network of miRNA functional similarity. Then, Xuan *et al*.^17^ developed a reliable prediction method based on random walk, they assigned different weights to transition matrix of miRNAs depending on whether they are associated with given diseases to exploit the prior information of nodes and the various ranges of topologies. And they extended the walk on a miRNA-disease bipartite network to predict candidates miRNAs, specially for the diseases without any known related miRNAs. Furthermore, Chen *et al*.^18^ developed a novel prediction method named WBSMDA for inferring miRNA-disease based on integrating miRNA functional similarity, disease semantic similarity, the known miRNA-disease associations, and the Gaussian interaction profile kernel similarity into heterogeneous network. WBSMDA not only could deal with new diseases without any known associated miRNAs, but also could handle new miRNAs without any known associated disease. In 2016, Zeng *et al*.^19^ conducted a review on methods for predicting disease and miRNA associations based on biological interaction networks. After detailed comparing these methods, they pointed out the current challenges in predicting disease and miRNA correlations. Liu *et al*.^20^ further proposed a method to explore potential miRNAs related to diseases by integrating multiple biology data in 2017. In recent years, recommendation system algorithms have been successfully applied in many fields. Chen *et al*.^21^ presented a hybrid approach for miRNA-disease association prediction (HAMDA) method based on hybrid recommendation methods, which combined available biology data and network-based inference methods. However, just like the above mentioned methods, they only prioritized miRNAs by utilizing the same layers neighbor nodes of miRNAs and diseases rather than making use of the different structural and topological characteristics among subgraphs of heterogeneous networks. Luo *et al*.^22^ proposed a novel effective prediction model that use unbalanced bi-random walk to improve performance of prediction. They fully exploited the different topological and structural of miRNA similarity networks and disease similarity network. This method improved prediction performance, but ignored the prior information and the respective topological structural of bipartite network. Zeng *et al*.^23^ found that heterogeneous miRNA-disease networks perform better on prediction than single disease similarity networks, miRNA similarity networks, and the known disease-gene association networks in 2018. So they adopted a method of structural perturbation to improve the prediction accuracy of miRNA-disease association.

We believe that the topological and structural features of heterogeneous network contain important information which is useful for discovering more reliable miRNA-disease associations. In present work, we develop an efficient computational method based on hybrid recommendation approach and unbalanced bi-random walk, called BRWHNHA (Bi-random Walk on Heterogeneous Network based on Hybrid Approach), which exploits the characteristic of nodes and the topological structural of the known miRNA-disease association by using hybrid recommendation approach, and taken advantage of the different topological structural between similarity networks of miRNA and disease by adopting bi-random walk on heterogeneous network. The hybrid recommendation algorithm adds some virtual edges to heterogeneous networks by calculating the transition matrix of bipartite network, so that the unbalanced bi-random walk on the new heterogeneous network can find potential miRNAs related to diseases more efficiently. To validate the prediction ability of BRWHNHA, we adopted five-fold cross-validation and compared BRWHNHA with MIDPE^17^, HAMDA^21^, and BRWH^22^. The average AUC is 2.13%, 0.69%, and 2.20% higher than the three methods. The case studies on lung neoplasms and prostatic neoplasms, and in the top 50 predicted associations, there are 49 and 46 real associations, respectively. It further demonstrates the ability of BRWHNHA in discovering potential miRNAs associated with disease.

## Results

To evaluate the prediction effectiveness of BRWHNHA in exploring undiscovered association between miRNAs and diseases, we compared BRWHNHA with MIDPE^17^, HAMDA^21^, and BRWH^22^ by five-fold cross-validation with repeating 100 times on the dataset obtained by Luo and Xiao^22^. For a given disease, we randomly divided the known-related miRNAs into five subsets with equal size. For each round, we used one subset as testing set and other four subsets as training set. After 5 rounds, we calculated the average AUC value. In order to reduce false positive, we recalculated miRNAs similarity and obtained a bran-new similarity matrix in each round of prediction. Then we calculated the probability of association between the given disease and miRNAs by BRWHNHA. Finally, all candidate miRNAs were ranked by association probability. The higher the miRNAs in testing set were ranked, the better the performance. As the most of diseases only have a few association with miRNAs that have been proved, the performance of the prediction methods can not be accurately evaluated. Hence we only tested the 22 diseases associated with at least 60 miRNAs as Luo and Xiao^22^. We only showed recall-precision curve of breast neoplasms and lung neoplasms. In addition, we analyzed effect of parameters on performance of BRWHNHA.

### Performance evaluation

In this study, the novelty of BRWHNHA was to calculate the transition probability matrix of bipartite network by using hybrid recommendation algorithm, and then a bi-random walk on heterogeneous network based on hybrid approach was adopted. The average AUC is 83.55% without using hybrid recommendation algorithm, which was 2.14% less than using hybrid recommendation algorithm. Therefore, it is important to construct the transition probability matrix by hybrid recommendation algorithm. The prediction accuracy was actually improved by exploring the prior information and topological structure of bipartite networks. The same heterogeneous network was used on MIDPE^17^, HAMDA^21^ and BRWH^22^. The best parameters of *α* = 0.9 and *γ* = 0.8 for MIDPE, *σ* = 0.7 and *ρ* = 0.8 for HAMDA *λ* = 0.6, *α* = 0.4, *r* = 2, *l* = 1 for BRWH were adopted as reported in original papers.

As illustrated in Table 1, the average AUC values of MIDPE, HAMDA, BRWHA and BRWHNHA in 22 diseases are 83.55%, 85.00%, 83.49% and 85.69% respectively. BRWHNHA performed the best with AUC 2.13%, 0.69% and 2.20% higher than other three methods. Moreover, BRWHNHA is superior to MIDPE and BRWH in all measurements for 22 diseases. Although HAMDA achieves higher AUC than BRWHNHA in 7 out of 22 diseases, but BRWHNHA obtains better performance in most of diseases. Since HAMDA repeatedly uses the known miRNAs-disease association data in the measurement of miRNAs similarity, it maybe overestimate the results. ROC curves of BRWHNHA and other three methods corresponding to the maximum AUC value in five-fold cross-validation at 100 times have shown in Supplementary Fig. S1 in Additional file.

**Table 1:** Predicting outcomes for MIDPE, HAMDA, BRWH and BRWHNHA by the five-fold cross-validation.

Diseases name	Number of related miRNAs	The average AUC			
		MIDPE^17^	HAMDA^21^	BRWH^22^	BRWHNHA
Breast Neoplasms	202	0.813	0.821	0.812	**0**.**855**
Carcinoma, Hepatocellular	214	0.777	0.791	0.776	**0**.**805**
Carcinoma, Non-Small-Cell Lung	95	0.859	0.867	0.857	**0**.**874**
Carcinoma, Renal Cell	107	0.814	0.822	0.812	**0**.**828**
Carcinoma, Squamous Cell	80	0.874	**0**.**886**	0.875	0.882
Colonic Neoplasms	78	0.855	0.866	0.853	**0**.**872**
Colorectal Neoplasms	147	0.819	0.838	0.824	**0**.**848**
Endometriosis	62	0.815	**0**.**850**	0.814	0.841
Esophageal Neoplasms	74	0.794	0.793	0.793	**0**.**825**
Glioblastoma	96	0.805	0.825	0.801	**0**.**839**
Glioma	71	0.868	**0**.**878**	0.863	0.874
Head and Neck Neoplasms	64	0.872	**0**.**886**	0.870	0.881
Heart Failure	120	0.802	**0**.**810**	0.800	0.807
Leukemia, Myeloid, Acute	64	0.857	**0**.**868**	0.852	0.858
Lung Neoplasms	132	0.906	0.919	0.906	**0**.**924**
Medulloblastoma	62	0.801	0.807	0.798	**0**.**811**
Melanoma	141	0.823	0.838	0.825	**0**.**849**
Ovarian Neoplasms	114	0.866	0.902	0.887	**0**.**903**
Pancreatic Neoplasms	99	0.905	0.907	0.902	**0**.**911**
Prostatic Neoplasms	118	0.833	0.857	0.831	**0**.**864**
Stomach Neoplasms	173	0.782	0.802	0.783	**0**.**832**
Urinary Bladder Neoplasms	92	0.842	**0**.**867**	0.833	**0**.**867**

**Figure 1:**
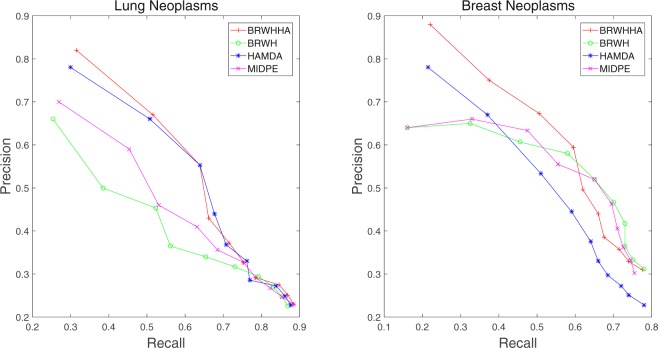
Recall-precision curves of breast neoplasm and lung neoplasm by five-fold cross-validation.

In Fig. 1, we compared BRWHNHA with other three methods in the recall-precision curves of breast neoplasms and lung neoplasms based on five-fold cross-validation. The precision-recall curve was obtained by measuring recall and precision at positions of top *k* (*k* = 10, 20, …, 100). The results show that our method achieves the highest precision and recall in the top 20. Moreover, with the increase of *k* value, the precision of BRWHNHA decreases, but the recall increases. It suggestes that the associations ranked in top position have higher probability of being potential miRNA-disease associations. We also compared the statistical significance of the difference in predictive ability between BRWHNHA and other three methods by paired *t*-tests. The *P*-values are listed in Table 2. Obviously, BRWHNHA achieves better performance than MIDPE, HAMDA, BRWHA at the significance level of 0.05.

**Table 2:** Pairwise comparison between BRWHNHA and another method by paired t-test on the AUC of prediction.

Method	MIDPE^17^	HAMDA^21^	BRWH^22^
P-value	2.41E-07	1.71E-02	4.00E-08

We also compared our method with SPM on the dataset used by Zeng *et al*.^23^. We found that our method performs slightly better than SPM on five subsets with equal size in five-fold cross-validation in most cases (comparison results are not shown here).

### Effect of parameters in BRWHNHA

There are four parameters *λ*, *α*, *r* and *l* explored in our method. The parameter *λ* is the hybridization parameter to mediate between HeatS algorithm and ProbS algorithm different kinds of resource distribution processes, and parameter *α* plays the role to control the consistence between the predicted candidate miRNA-disease associations and the known associations. The parameters of *r* and *l* are the numbers of maximal random walk steps in miRNA similarity network and disease similarity network, respectively. We set various values of *λ* and *α* ranging from 0 to 1, the step length was 0.1. *r* and *l* were taken to be between 0 step to 5 steps, the step length was 1. Then, we calculated average AUC in the framework of five-fold cross-validation. Table 3 shows the effects of *λ*, *α*, *r* and *l* on the cross validation result in miRNA-disease association dataset. It can be observed that BRWHNHA achieves the best performance, when *λ* = 0.6, *α* = 0.4, *r* = 2, *l* = 1.

**Table 3:** Effects of parameters *λ*, *α*, *r*, *l* on prediction performance of BRWHNHA.

*α* = 0.4, *r* = 2, *l* = 1		*λ* = 0.6, *r* = 2, *l* = 1	
*λ*	Average AUC	*α*	Average AUC
0	0.748654	0	0.804929
0.1	0.814260	0.1	0.844848
0.2	0.844524	0.2	0.853413
0.3	0.852676	0.3	0.856242
0.4	0.855220	0.4	**0.856818**
0.5	0.856081	0.5	0.855095
0.6	**0.856818**	0.6	0.853919
0.7	0.856418	0.7	0.850477
0.8	0.856146	0.8	0.848039
0.9	0.855824	0.9	0.847697
1	0.855415	1	0.847478
***λ*** = **0**.**6**, ***α*** = **0**.**4**, ***l*** = **1**		***λ*** = **0**.**6**, ***α*** = **0**.**4**, ***r*** = **2**	
***r***	**Average AUC**	***l***	**Average AUC**
0	0.846267	0	0.856710
1	0.852731	1	**0.856818**
2	**0.856818**	2	0.853561
3	0.856814	3	0.846865
4	0.856555	4	0.846626
5	0.856371	5	0.846505

## Case study

To further validate efficiency of BRWHNHA for discovering the potential associations between miRNAs and diseases, we conducted two case studies of Lung neoplasms and Prostatic neoplasms here. All known miRNA-disease associations released in June 2014 were regarded as training sets, and the set of candidate associations formed by all other associations. The prediction results of Lung neoplasms and Prostatic neoplasms were confirmed based on relevant literatures and two important public database: dbDEMC^24^ and MiR2Disease^25^.

Lung cancer is one of the malignant tumors with the highest morbidity and mortality, and it is the greatest health and life threat to human. Over the past 50 years, many countries have reported significant increases in the incidence and mortality of lung cancer. The first 50 predicted miRNA associated with lung cancer were shown in Table 4. As a result, among the top 20 and 50 potential Lung neoplasms associated miRNAs, 20 and 49 were confirmed by dbDEMC database, MiR2Disease and literature. Though there is no database or literature that proved the miRNA (hsa-mir-200) relevance to lung neoplasms, the mir-200 family, which includes 5 members (miRNA-200a, miRNA-200b, miRNA-200c, miRNA-429, and miRNA-141), is associated with Lung neoplasms in dbDEMC, so we have reason to believe that it is related to the disease. In addition, we also listed the potential miRNAs from top 51 to top100 (Supplementary Table S2 in Additional file 1).

**Table 4:** The first 50 potential miRNAs associated with lung neoplasms predicted by BRWHNHA.

Rank	miRNAs	Evidence	Rank	miRNAs	Evidence
1	hsa-mir-20b	DB	26	hsa-mir-302f	DB
2	hsa-mir-15b	DB	27	hsa-mir-1258	DB
3	hsa-mir-34b	DB	28	hsa-mir-1305	DB
4	hsa-mir-21	DB, MD	29	hsa-mir-140	DB, MD
5	hsa-mir-200b	DB, MD	30	hsa-mir-106a	DB, MD
6	hsa-mir-29b	DB, MD	31	hsa-mir-219	DB, MD
7	hsa-mir-146a	DB, MD	32	hsa-mir-1827	PMID:21676885
8	hsa-mir-7i	DB	33	hsa-mir-675	DB
9	hsa-mir-1236	DB	34	hsa-mir-485	DB
10	hsa-mir-30e	DB, MD	35	hsa-mir-105	DB
11	hsa-let-200a	DB, MD	36	hsa-mir-92b	DB
12	hsa-mir-885	DB	37	hsa-mir-1323	DB
13	hsa-let-147b	DB	38	hsa-mir-135a	DB
14	hsa-let-10a	DB	39	hsa-mir-98	DB, MD
15	hsa-mir-198	DB, MD	40	hsa-mir-137	DB
16	hsa-let-100	DB	41	hsa-mir-27a	DB
17	hsa-mir-212	DB, MD	42	hsa-mir-235	DB
18	hsa-mir-181d	DB	43	hsa-mir-450b	DB
19	hsa-mir-217	DB	44	hsa-mir-495	DB
20	hsa-mir-204	DB, MD	45	hsa-mir-18a	DB, MD
21	hsa-mir-374b	DB	46	hsa-mir-1915	DB
22	hsa-mir-133a	DB	47	hsa-mir-101	DB, MD
23	hsa-mir-200	Unconfirmed	48	hsa-mir-23a	DB
24	hsa-mir-106b	DB	49	hsa-mir-1207	DB
25	hsa-mir-194	DB	50	hsa-mir-337	DB

*The databases dbDEMC and mir2disease are respectively represented as DB and MD.

Prostate neoplasms is an important malignant tumor in male patients. There are usually no clinical symptoms in the early stage. Currently, most of the patients admitted by prostate neoplasms are in the late stage^26^. Therefore, early diagnosis is an urgent problem. There are many evidences that have confirmed a link between miRNA and prostate neoplasms, and it could be therapeutically useful for the treatment of prostate neoplasms by regulating the expression of related miRNAs^27,28^. As a result of the case study for prostate neoplasms, 18 out of the top-20 and 46 out of the top-50 predicted miRNAs of prostate neoplasms were verified by dbDEMC, MiR2Disease and literature (shown in Table 5). However, hsa-mir-302f, hsa-mir-1915, hsa-mir-4257 and hsa-mir-1286 are not included in dbDEMC, MiR2Disease and literature. We also listed the potential miRNAs from top 51 to top100 (Supplementary Table S3 in Additional file 1).

**Table 5:** The first 50 potential miRNAs associated with prostate neoplasms predicted by BRWHNHA.

Rank	miRNAs	Evidence	Rank	miRNAs	Evidence
1	hsa-mir-154	DB	26	hsa-mir-137	PMID:26461474
2	hsa-mir-189	DB	27	hsa-mir-134	DB
3	hsa-mir-19a	DB	28	hsa-mir-30a	DB, MD
4	hsa-mir-29b	DB, MD	29	hsa-mir-138	PMID:28741117
5	hsa-mir-21	DB, MD	30	hsa-mir-302a	DB
6	hsa-mir-199b	DB, MD	31	hsa-mir-450b	DB
7	hsa-mir-141	DB, MD	32	hsa-mir-495	DB
8	hsa-mir-10a	DB, MD	33	hsa-mir-26a	DB, MD
9	hsa-mir-181	DB	34	hsa-mir-20a	DB, MD
10	hsa-mir-885	DB	35	hsa-mir-139	DB
11	hsa-let-7d	DB, MD	36	hsa-mir-107	DB
12	hsa-mir-149	DB, MD	37	hsa-mir-148b	DB
13	hsa-let-7e	DB	38	hsa-mir-2355	DB
14	hsa-let-7f	DB, MD	39	hsa-mir-302b	DB
15	hsa-mir-195	DB, MD	40	hsa-mir-337	DB
16	hsa-let-7g	DB, MD	41	hsa-mir-624	DB
17	hsa-mir-302f	Unconfirmed	42	hsa-mir-214	DB, MD
18	hsa-mir-23b	DB, MD	43	hsa-mir-205	DB, MD
19	hsa-mir-199a	DB, MD	44	hsa-mir-1286	Unconfirmed
20	hsa-mir-1915	Unconfirmed	45	hsa-mir-340	DB
21	hsa-mir-1258	DB	46	hsa-mir-1275	DB
22	hsa-mir-675	DB	47	hsa-mir-136	DB
23	hsa-mir-4257	Unconfirmed	48	hsa-mir-218	DB, MD
24	hsa-mir-18a	DB	49	hsa-mir-498	DB, MD
25	hsa-mir-101	DB, MD	50	hsa-mir-301b	PMID:29744254

*The databases dbDEMC and mir2disease are respectively represented as DB and MD.

## Conclusion

Taking full account of the different topological and structural characteristics of heterogeneous network is a very challenging and meaningful task in prioritizing potential disease-related miRNAs. In this paper, we first adopted an effective measurement, which is suitable for miRNAs and diseases without known miRNA-disease associations, to estimate the similarity of miRNAs and diseases. Then, we presented a BRWHNHA method based on hybrid recommendation algorithm and unbalanced bi-random walk to predict potential diseases associated miRNAs. We made full use of the prior information and topological structural by calculating the transition probability matrix of bipartite network in using hybrid recommendation algorithm, in addition, we fully exploited the topologies and structures of miRNA similarity network(MMS) and disease similarity network(DDS) in the different lever by adopting unbalanced bi-random walk on heterogeneous network. To assess the performance of BRWHNHA, we compared BRWHNHA with MIDP, HAMDA and BRWH on the dataset obtained by Luo and Xiao^22^. The results indicate that BRWHNHA has the best prediction ability among these methods, the average AUC was 2.13%, 0.69% and 2.20% higher than MIDP, HAMDA and BRWH, respectively. Furthermore, case studies on lung neoplasms and prostatic were employed to further identify the performance evaluation of BRWHNHA, which the top 49 out of 50 and 46 out of 50 predicted miRNA-disease associations respectively were confirmed by recently published literature and databases of dbDEMC and MiR2Disease. The results show that BRWHNHA can be used as an effective and important method to explore the potential association between miRNAs and diseases.

Nevertheless, there is a limitation on our BRWHNHA that should be improved in future study. That is there are many parameters need to be set in this method. So a more effective method need be adopted to find the optimal parameters.

## Methods

### The measurement of disease semantic similarity and miRNA functional similarity

**Figure 2:**
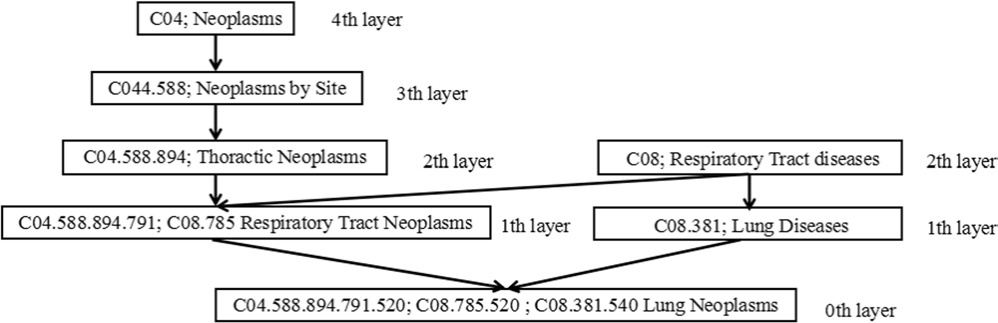
Hierarchical DAG graph of Lung neoplasms.

As described in the category C of MeSH descriptor, the disease relationships can be regarded as a directed acyclic graph structure. The disease K can be represented as *DAG*(*K*) = (*K*, *T*(*K*), *E*(*K*))^22^, where *T*(*K*) represents the set of all the ancestor nodes of disease *K* and disease *K*, *E*(*K*) represents the set of all direct edges from parent nodes to child nodes in the subgraph, as shown in Fig. 2. For two diseases *d*_*i*_ and *d*_*j*_, the disease semantic similarity measurement *DSS*(*d*_*i*_, *d*_*j*_) is defined by Luo and Xiao^22^.

Based on the assumption that miRNAs with similar functions are more likely to be associated with similar diseases and vice versa^29,30^, the miRNA function similarity measurement *MFS*(*m*_*i*_, *m*_*j*_) for two miRNAs *m*_*i*_ and *m*_*j*_ is adopted, which proposed by Wang *et al*.^31^.

### Gaussian interaction profile kernel similarity

The Gaussian interaction profile kernel similarity also is based on the assumption that miRNAs with similar functions are more likely to be associated with similar diseases and vice versa. Let $A={({a}_{ij})}_{{n}_{m}\times {n}_{d}}$ be the adjacency matrix of MD, and *n*_*m*_ denotes the number of miRNAs and *n*_*d*_ the number of diseases, respectively. The Gaussian interaction profile kernel similarity is calculated by the known miRNA-disease associations^21^, so let *IP*(*d*_*i*_) binary vector indicate whether disease *d*_*i*_ is associated with each miRNA, in other words, *IP*(*d*_*i*_) is the *i*th column of *A*. The Gaussian interaction profile kernel similarity between two diseases *d*_*i*_ and *d*_*j*_ is calculated as:

1 $$DGS({d}_{i},{d}_{j})=exp(\,-\,{r}_{d}\parallel IP({d}_{i})-IP({d}_{j}){\parallel }^{2})$$

where ${r}_{d}={r^{\prime} }_{d}/(\tfrac{1}{{n}_{d}}\,{\sum }_{i=1}^{{n}_{d}}\,\parallel IP({d}_{i}){\parallel }^{2})$ is the kernel bandwidth and ${r}_{d}^{^{\prime} }$ is a new bandwidth parameter (e.g. (${r}_{d}^{^{\prime} }=1$ as^32,33^) to normalize *r*_*d*_.

For two miRNAs *m*_*i*_ and *m*_*j*_, the Gaussian interaction profile kernel similarity is calculated as:

2 $$MGS({m}_{i},{m}_{j})=exp(\,-\,{r}_{m}\parallel IP({m}_{i})-IP({m}_{j}){\parallel }^{2})$$

where ${r}_{m}={r^{\prime} }_{m}/(\tfrac{1}{{n}_{m}}\,{\sum }_{i=1}^{{n}_{m}}\,\parallel IP({m}_{i}){\parallel }^{2})$ is the kernel bandwidth and ${r}_{m}^{^{\prime} }$ is a new bandwidth parameter to normalize *r*_*m*_.

### Integrated similarity for miRNAs and diseases

A new disease similarity matrix can be obtained by integrated disease semantic similarity and the disease Gaussian interaction profile kernel similarity^21^. For two diseases *d*_*i*_ and *d*_*j*_, the new diseases similarity can be defined as follows:

3 $${D}_{S}({d}_{i},{d}_{j})=\{\begin{array}{ll}DSS({d}_{i},{d}_{j}), & DSS({d}_{i},{d}_{j})\ne 0\\ DGS({d}_{i},{d}_{j}), & {\rm{otherwise}}\end{array}$$

The integrated similarity between miRNAs *m*_*i*_ and *m*_*j*_ can be defined as follows:

4 $${M}_{S}({m}_{i},{m}_{j})=\{\begin{array}{ll}MFS({m}_{i},{m}_{j}), & MFS({m}_{i},{m}_{j})\ne 0\\ MGS({m}_{i},{m}_{j}), & {\rm{otherwise}}\end{array}$$

### Hybrid recommendation algorithm

A binary network *MD*(*M*, *D*, *E*) is constructed by experimentally confirmed miRNA-disease association, where *D* represents all diseases nodes, *M* represents all miRNAs nodes, and *E* represents all edges in MD. The adjacency matrix *A* is defined as follows:

5 $${a}_{ij}=\{\begin{array}{ll}1, & {m}_{i}\,{\rm{is}}\,{\rm{associated}}\,{\rm{with}}\,{d}_{j}\\ 0, & {\rm{otherwise}}\end{array}$$

**Figure 3:**
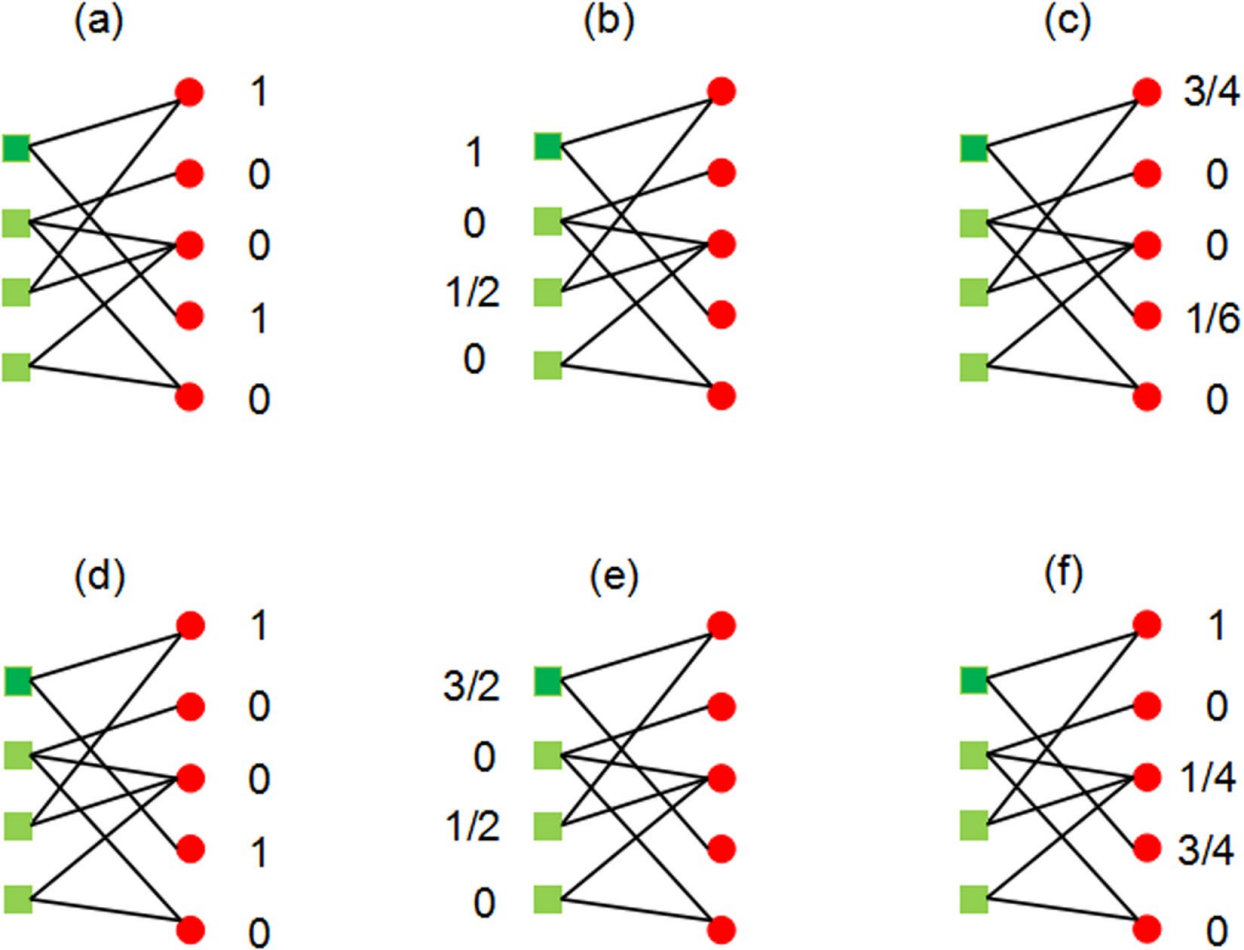
The HeatS (**a**–**c**) and ProbS (**d**–**f**) algorithms at work on the bipartite miRNA-disease network. Disease are shown as green squares and dark green squares is a given disease, miRNAs are shown as red circles.

Zhou *et al*.^34^ proposed the hybrid recommendation algorithm, which combined the heat spreading (HeatS) algorithm and probabilistic spreading (ProbS) algorithm by incorporating the hybridization parameter *λ* to balance the accuracy of HeatS and the diversity of ProbS. For a given disease, HeatS and ProbS both work by assigning miRNA an initial resource represented by the vector *f* (where *f*_*i*_ is the resource possessed by miRNA *m*_*i*_), which was redistributed though the transformation $f^{\prime} =W\,\ast \,f$. The miRNA that possess more resource is more likely associated the given disease. In Fig. 3, the visualization process of HeatS algorithm and ProbS algorithm is presented.

HeatS is defined as follows:

6 $${W}_{ij}^{H}=\frac{1}{k({m}_{i})}\,{\sum }_{l=1}^{{n}_{d}}\,\frac{{a}_{il}{a}_{jl}}{k({d}_{l})}$$

7 $$f^{\prime} ={W}^{H}\ast f$$

ProbS is defined as follows:

8 $${W}_{ij}^{P}=\frac{1}{k({m}_{j})}\,{\sum }_{l=1}^{{n}_{d}}\,\frac{{a}_{il}{a}_{jl}}{k({d}_{l})}$$

9 $$f^{\prime} ={W}^{P}\ast f$$

Hybrid recommendation algorithm is defined as follows:

10 $${W}_{ij}^{H+P}=\frac{1}{k{({m}_{i})}^{1-\lambda }k{({m}_{j})}^{\lambda }}\,\sum _{l=1}^{nd}\,\frac{{a}_{il}{a}_{jl}}{k({d}_{l})}$$

11 $$f^{\prime} ={W}^{H+P}\ast f$$

*k*(*x*) denotes the degree of nodes *x* in bipartite graph *MD*(*M*, *D*, *E*).

### Our method BRWHNHA

**Figure 4:**
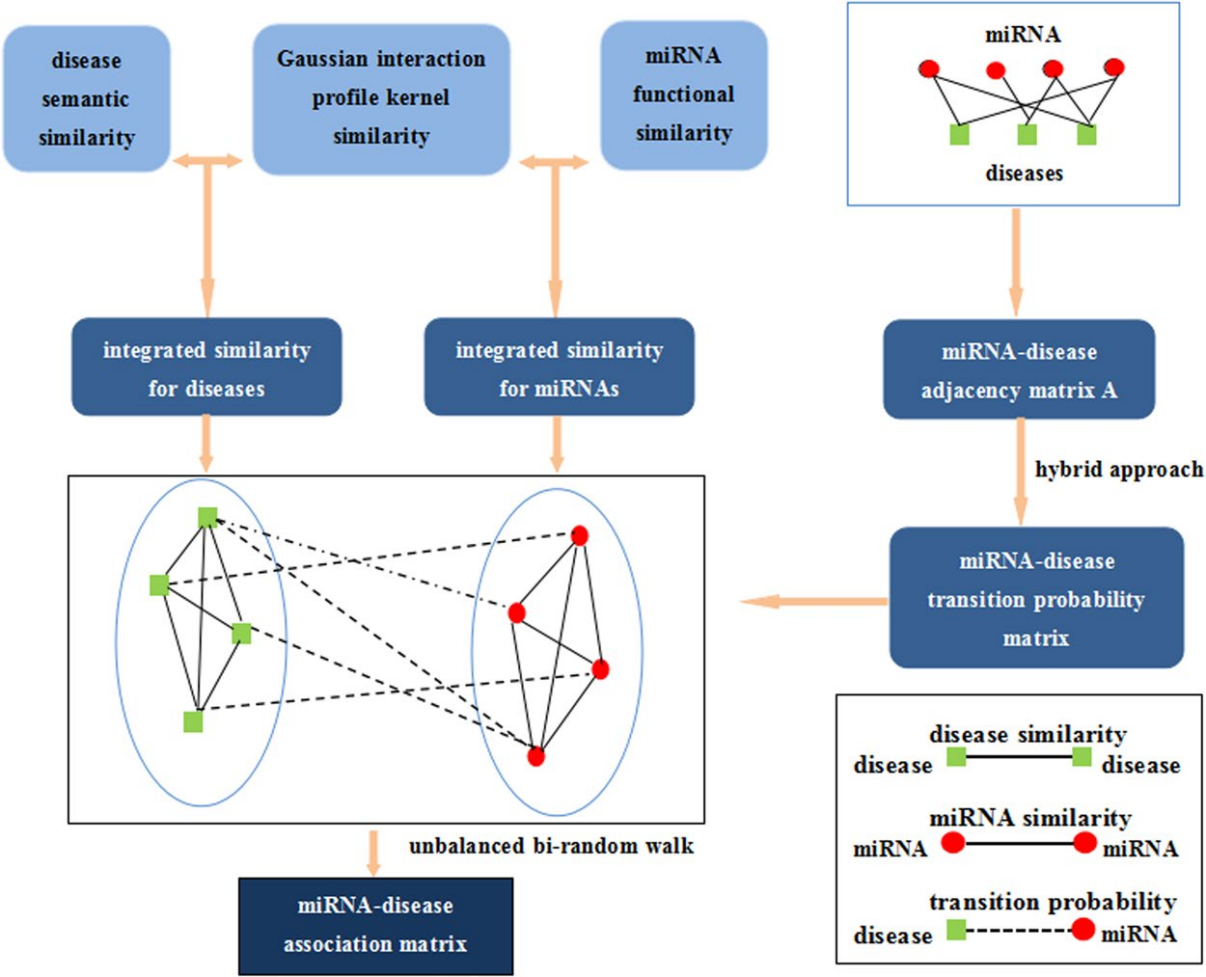
Flowchart of potential miRNA-disease association prediction based on the computational model of BRWHNHA.

In this paper, we present a BRWHNHA method based on hybrid recommendation algorithm and unbalance bi-random walk to predict potential diseases associated miRNAs. Luo *et al*.^22^ found that most of the nodes in DDS and MMS are isolated, and the sparsity of disease semantic similarity and miRNA functional similarity effect the prediction performance. To overcome this disadvantages in data, the similarity is estimated for each disease pair via integrating disease semantic similarity and disease Gaussian interaction profile kernel similarity, as well as miRNA pair is estimated via integrating miRNA function similarity and miRNA Gaussian interaction profile kernel similarity. Then, the bipartite miRNA-disease network (MD) is constructed, where edges in the miRNA-disease network are the known associations between miRNAs and diseases that were released by HMDD in June 2014. The transition probability matrix of MD is obtained by using hybrid recommendation algorithm in bipartite networks. Then, unbalance bi-random walk is carried out in heterogeneous network that includes DDS, MMS and MD. Finally, for a given disease, all candidate miRNAs will be ranked according to transition probability matrix, and the higher the rank, the more likely it is to be associated with the given disease. Flowchart of potential miRNA-disease association prediction based on the computational model of BRWHNHA is shown in Fig. 4. The most important 2 steps is:

Step 1 (Calculate the transition probability matrix of DDS, MMS and MD): The transition probability matrix $M={(M(i,j))}_{{n}_{m}\times {n}_{m}}$ of MMS is constructed as:

12 $$M(i,j)=\{\begin{array}{ll}\tfrac{{M}_{S}(i,j)}{{\sum }_{k=1}^{{n}_{m}}\,{M}_{S}(k,j)}, & \sum _{k=1}^{{n}_{m}}\,{M}_{S}(k,j)\ne 0\\ 0, & {\rm{otherwise}}\end{array}$$

Similarly, $D{(D(i,j))}_{{n}_{d}\times {n}_{d}}$ is the transition probability matrix of the DDS:

13 $$D(i,j)=\{\begin{array}{ll}\tfrac{{D}_{S}(i,j)}{{\sum }_{k=1}^{{n}_{d}}\,{D}_{S}(k,j)}, & \sum _{k=1}^{{n}_{d}}\,{D}_{S}(k,j)\ne 0\\ 0, & {\rm{otherwise}}\end{array}$$

Based on hybrid recommendation algorithm, the miRNA node *m*_*i*_ is assigned an initial lever of resource *f*(*m*_*i*_) = 1, *or* 0 depending on whether the miRNA is associated with given disease. All the resource of miRNA nodes redistributed via the transition matrix of hybrid recommendation algorithm, and transition probability matrix of MD is calculated as:

14 $${P}_{A}={W}^{H+P}\ast A.$$

Step 2 (Implement unbalance bi-random walk in heterogeneous network): Because of the different topological characteristics between MMS and DDS, in these two networks, we introduce two parameters of *l* and *r* as the biggest step random walk on MMS and DDS.

15 $$MMS:{P}_{{t}_{ \mbox{-} M}}=(1-\alpha )\ast M\ast {P}_{t-1}+\alpha \ast {P}_{A}$$

16 $$DDS:{P}_{{t}_{ \mbox{-} D}}=(1-\alpha )\ast {P}_{t-1}\ast D+\alpha \ast {P}_{A}$$

17 $${P}_{t}=\{\begin{array}{ll}\tfrac{{P}_{{t}_{ \mbox{-} M}}+{P}_{{t}_{ \mbox{-} D}}}{2} & t\le r,t\le l\\ {P}_{{t}_{ \mbox{-} M}}, & t\le r,t > l\\ {P}_{{t}_{ \mbox{-} D}}, & t > r,t\le l\end{array}$$

*α* denotes a decay factor ranging from 0 and 1. The matrix *P*_*A*_ is used to control the prior probability of the iterative process and is the transition probability matrix of the bipartite network *G* obtained by the recommendation algorithm. *P*_*A*_ is a transition probability matrix, and *P*_0_ = *P*_*A*_/*sum*(*P*_*A*_). After several iterations, *P*_*t*_ is the steady-state probability matrix between miRNAs and diseases. For a given disease, we ranked all the candidate miRNAs based on the probability. In BRWHNHA algorithm, we effectively utilize the topological information of heterogeneous networks, including: MMS, DDS and MD.

## Supplementary information

Additional file 1

Additional file 2

Additional file 3

Additional file 4

## Data Availability

All data generated or analyzed during this study are included in this article [Additional file 2, Additional file 3, Additional file 4]. (The data we used was downloaded from the paper of Luo and Xiao^22^).
