# Supplementary material for: Inferring microRNA-disease association by hybrid recommendation algorithm and unbalanced bi-random walk on heterogeneous network
Source: Sci Rep. 2019 Feb 21;9:2474. doi: 10.1038/s41598-019-39226-x (PMC6385311; doi:10.1038/s41598-019-39226-x)
Supplement: Supplementary file 1 — Additional file 1 [file 41598_2019_39226_MOESM1_ESM.pdf]

Inferring microRNA-disease association by hybrid recommendation algorithm and unbalanced bi-random walk on heterogeneous network

Dong-Ling Yu1, Yuan-Lin Ma1,+, and Zu-Guo Yu1,2,*

1School of Mathematics and computational Science, Xiangtan University, Hunan 411105, China.
2School of Electrical Engineering and Computer Science, Queensland University of Technology, Q4001, Australia.
*Corresponding author: yuzuguo@aliyun.com
+Joint first author

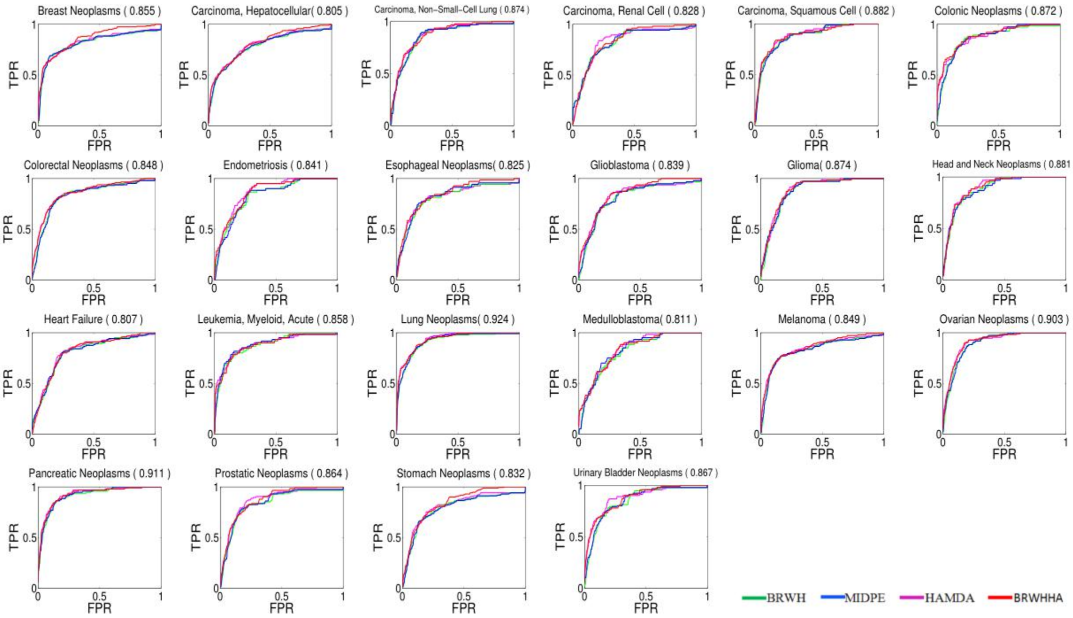

Supplementary Figure S1 : ROC curves of BRWHNHA and other methods for five-fold cross validation.

Supplementary Table S2: The top 50 to 100 potential miRNAs associated with lung neoplasms predicted by BRWHNHA .

Rank	miRNA	Evidence	Rank	miRNA	Evidence
51	hsa-mir-134	DB	76	hsa-mir-130b	DB
52	hsa-mir-377	DB	77	hsa-mir-624	Unconfirmed
53	hsa-mir-331	DB	78	hsa-mir-136	DB
54	hsa-mir-498	DB	79	hsa-mir-301b	DB
55	hsa-mir-151	DB	80	hsa-mir-326	DB
56	hsa-mir-20a	DB, MD	81	hsa-mir-205	DB, MD
57	hsa-mir-139	DB, MD	82	hsa-mir-196b	DB
58	hsa-mir-2861	Unconfirmed	83	hsa-mir-628	DB
59	hsa-mir-26a	DB, MD	84	hsa-mir-363	DB
60	hsa-mir-1275	DB	85	hsa-mir-130a	DB, MD
61	hsa-mir-30a	DB, MD	86	hsa-mir-214	DB, MD
62	hsa-mir-151b	DB	87	hsa-mir-338	DB, MD
63	hsa-mir-193b	DB	88	hsa-mir-27b	DB, MD
64	hsa-mir-95	DB, MD	89	hsa-mir-143	DB, MD
65	hsa-mir-138	DB	90	hsa-mir-365b	DB
66	hsa-mir-302a	DB	91	hsa-mir-372	DB
67	hsa-mir-190b	DB	92	hsa-mir-92a	DB
68	hsa-mir-4257	Unconfirmed	93	hsa-mir-451b	Unconfirmed
69	hsa-mir-7	DB, MD	94	hsa-mir-185	DB
70	hsa-mir-148b	DB	95	hsa-mir-493	DB
71	hsa-mir-302b	DB	96	hsa-mir-448	DB
72	hsa-mir-1286	DB	97	hsa-mir-3196	Unconfirmed
73	hsa-mir-203	DB, MD	98	hsa-mir-206	DB
74	hsa-mir-99a	DB, MD	99	hsa-mir-32	DB, MD
75	hsa-mir-30c	DB	100	hsa-mir-539	DB

Supplementary Table S3: The top 50 to 100 potential miRNAs associated with prostate neoplasms predicted by BRWHNHA .

Rank	miRNA	Evidence	Rank	miRNA	Evidence
51	hsa-mir-365b	DB	76	hsa-mir-422a	DB
52	hsa-mir-371a	Unconfirmed	77	hsa-mir-376b	DB
53	hsa-mir-196b	DB	78	hsa-mir-342	DB
54	hsa-mir-181b	DB, MD	79	hsa-mir-27b	DB
55	hsa-mir-363	DB	80	hsa-mir-186	DB
56	hsa-mir-185	DB	81	hsa-mir-371	DB
57	hsa-mir-326	DB	82	hsa-mir-375	DB, MD
58	hsa-mir-181c	DB	83	hsa-mir-520c	Unconfirmed
59	hsa-mir-365a	DB	84	hsa-mir-379	DB
60	hsa-mir-539	DB	85	hsa-mir-490	DB
61	hsa-mir-338	DB	86	hsa-mir-362	DB
62	hsa-mir-143	DB, MD	87	hsa-mir-423	DB
63	hsa-mir-206	DB	88	hsa-mir-637	DB
64	hsa-mir-302c	DB, MD	89	hsa-mir-516b	Unconfirmed
65	hsa-mir-449a	DB, MD	90	hsa-mir-502	DB
66	hsa-mir-2110	DB	91	hsa-mir-494	DB
67	hsa-mir-369	DB	92	hsa-mir-183	DB, MD
68	hsa-mir-944	DB	93	hsa-mir-492	Unconfirmed
69	hsa-mir-210	DB, MD	94	hsa-mir-512	DB
70	hsa-mir-92a	DB	95	hsa-mir-215	DB
71	hsa-mir-493	Unconfirmed	96	hsa-mir-325	Unconfirmed
72	hsa-mir-148a	DB	97	hsa-mir-519e	DB
73	hsa-mir-424	DB	98	hsa-mir-103a	Unconfirmed
74	hsa-mir-30d	DB	99	hsa-mir-129	DB
75	hsa-mir-4792	Unconfirmed	100	hsa-mir-520g	DB
